# Safety Assessment of *Bacteroides uniformis* CECT 7771 Isolated from Stools of Healthy Breast-Fed Infants

**DOI:** 10.1371/journal.pone.0145503

**Published:** 2016-01-19

**Authors:** M. Leonor Fernández-Murga, Yolanda Sanz

**Affiliations:** Microbial Ecology, Nutrition & Health Research Unit, Institute of Agrochemistry and Food Technology, National Research Council (IATA-CSIC), Valencia, Spain; Indian Institute of Science, INDIA

## Abstract

**Background:**

*Bacteroides uniformis* CECT 7771 is a potential probiotic strain, originally isolated from the stools of healthy breast-feed infants. The strain showed pre-clinical efficacy in a mouse obesity model. The objective of this study was to evaluate its potential toxicity and translocation ability after acute oral administration to mice.

**Methods and Findings:**

A safety study was conducted in immunocompetent and immunosuppressed C57BL-6 mice. Both mouse groups (n = 10 per group) were fed orally 2 x 10^9^ colony forming units (cfu)/day of *B*. *uniformis* CECT 7771 or placebo by gavage for 6 days. Throughout this time, feed and water intake and body weight were monitored. Afterwards, mice were sacrificed and biological samples were collected to analyze blood and urine biochemistry, inflammatory and immune markers; gut mucosal histology and bacterial translocation to peripheral tissues. The results demonstrated that acute ingestion of this *Bacteroides* strain had no adverse effects on the animals’ general health status or food intake, nor did it affect biochemical indicators of liver, kidney and pancreatic function or gut mucosal histology. Findings also demonstrated that administration did not lead to bacterial translocation to blood, liver or mesenteric lymph nodes. *B*. *uniformis* CECT 7771 also downregulated gene and protein expression (*iNOS* and *PPAR-γ*) and inflammatory cytokines induced by immunosuppression.

**Conclusions:**

The findings indicate that the acute oral consumption of *B*. *uniformis* CECT 7771 does not raise safety concerns in mice. Further studies in humans should be conducted.

## Introduction

Our understanding of the role played by the gut microbiota in health and disease has burgeoned thanks to the development of next generation sequencing techniques [1]. To identify associations with our health status and lifestyle, such techniques are being used to investigate the microbial structure and function in different body parts, as well as the relationships between the microbiota and the environment (e.g. diet, antibiotic intake, etc.), and with host genetic and phenotypic factors (age, diseases, etc.). This information will help us identify new bacterial species and strains, beyond those known as traditional probiotics (e.g. *Lactobacillus* spp., *Bifidobacterium* spp.), which may be used to reduce disease risk and optimize our physiological functions [2–4]. These new species and strains are normal inhabitants of the human intestinal tract but, unlike traditional probiotics [5–7], they lack a history of safe use as part of the human diet. Therefore, a specific safety assessment should guarantee their unambiguous safety status according to their intended use [8, 9]. These new bacterial isolates constituting the so-called next-generation of potential probiotics could, however, be considered as novel foods. Novel foods are defined as those that have not been substantially consumed in the EU prior to 15 May 1997 according to the Regulation 285/97/EC [10]. Based on this Regulation, the competent authority in the member state, the EFSA, and the European Commission (EC) make assessments to guarantee the safety of any food or food ingredient that has no history of safe use.

The genus *Bacteroides* represents between 20% and 40% of the human adult colonic bacteria, exceeding by far (by a factor of 10,000) *Lactobacillus* and *Bifidobacterium* [11]. Therefore, this genus and its specific components may presumably play an important role in the gut ecology and human health. However, potential exploitation of this genus as a probiotic is in the early stages.

Species and strains of the genus *Bacteroides* are known to have desirable traits and properties including the ability to (i) metabolize complex carbohydrates and generate short-chain fatty acids directly or via cross-feeding mechanisms (e.g., propionic and butyric acids), which may have positive effects on satiety and glucose metabolism [3–12]; (ii) outcompete pathogens by colonization resistance [13] and (iii) optimize the systemic Th1/Th2 balance and induce regulatory T cell differentiation, favoring tolerance and reducing autoimmune disorders according to murine studies [14]. Nevertheless, strains of the species *Bacteroides fragilis* may also produce toxins, potentially constituting opportunistic pathogens involved in infections and in chronic inflammatory disorders [15].

Abundance of the species *Bacteroides uniformis* is higher in breast-fed than in formula-fed infants [16]. In particular, the strain *B*. *uniformis* CECT 7771 was originally isolated from stools of healthy breast-fed infants [16]. It was selected for its ability to induce *in-vitro* anti-inflammatory cytokine production, and to ameliorate the metabolic and immune dysfunction of diet-induced obesity in mice. *B*. *uniformis* CECT 7771 reduced body weight gain, liver steatosis and liver cholesterol, and triglyceride levels in high-fat diet (HFD) fed mice. This strain also decreased serum glucose, insulin and leptin concentrations [3].

The aim of this study is to provide a preliminary evaluation of the safety and tolerability of *B*. *uniformis* CECT 7771 by short-term (acute) oral administration to normal and immunocompromised mice, assessing the general health status, bacterial translocation and different biochemical and immune markers.

## Materials and Methods

### Bacterial strain and culture conditions

*Bacteroides uniformis* CECT 7771 was isolated from stools of healthy infants [16] and deposited in the Spanish Culture Collection (CECT). The bacteria were grown in Schaedler medium without hemin broth at 37°C in microaerophilic conditions (AneroGen; Oxoid, Basingstoke, UK). Cells were harvested by centrifugation (6000 g for 15 min), washed twice in phosphate buffered saline (PBS, 130 mM sodium chloride, 10mM sodium phosphate, pH7.4). Cells were then re-suspended in 10% skimmed milk for animal trials. Aliquots of these suspensions were frozen in liquid nitrogen and stored at -80 C until use. After freezing and thawing, live cells numbers were determined by colony-forming unit (CFU) counting on Schaedler agar medium after 48 h incubation. For the strain tested, more than 90% cells were alive upon thawing and no significant differences were found during storage time (2 months). One fresh aliquot was thawed for every new experiment to avoid differences in culture viability.

### Acute toxicity study in immunocompetent and immunosuppressed mice

The acute toxicity study was basically performed according to Chenoll et al. [17] in 6–7 week-old male C57BL-6 mice (Harlan Laboratories, Barcelona, Spain) in specific pathogen-free conditions. During the adaptation period (7 days), five animals were housed in each stainless-steel cage in a temperature-controlled (23°C) room with a 12-h light/dark cycle and 40–50% relative humidity. All animals received a standard diet. Then, mice were randomly divided in four groups (n = 10 mice per group) as follows: 1) a group receiving a daily dose of placebo (10% [w/v] skimmed milk) (Control); 2) a group receiving a daily dose of 2x10^9^ CFU *B*. *uniformis* CECT 7771 by gavage (Control+B); 3) an immunodepressed group receiving a daily dose of placebo (IMM); and 4) an immunodepressed group receiving a daily dose of 2x10^9^ CFU *B*. *uniformis* CECT 7771 by gavage (IMM+B). Immunosuppression was induced by intraperitoneal administration of cyclophosphamide (250 mg/kg), 5 d prior to the intervention with the bacteroides strain and a second dose (40 mg/kg) after 3 days of intervention. The mice were kept inside containment units under positive pressure. Mortality and morbidity were recorded twice daily and individual body weights were recorded at the beginning and end of the trial. After the 6-day intervention, animals were anesthetized; blood was collected by aortic puncture from each mouse, which was then immediately killed by cervical dislocation. Large and small intestine, liver and mesenteric lymph nodes (MLN) were removed in sterile conditions, weighed and stored for different analyses as described below. All procedures involving animals were specifically approved by the ethics committee of the University of Valencia (Animal Production Section, Central Service of Support to Research [SCSIE], University of Valencia, Spain) and authorized by Dirección General de Agricultura, Ganadería y Pesca (Generalidad Valenciana” (approval ID A 1370964610964).

### Bacterial translocation

Bacterial translocation was assessed in samples of blood, liver and MLN. Samples were homogenized in buffered peptone water (1g/ml) and 100 μl of the resulting homogenates were inoculated in plates for bacterial counting, using Schaedler agar (Oxoid, UK) for *Bacteroides* and Wilkins- Chalgren anaerobe agar (Oxoid, agar) for total anaerobe quantification after incubation at 37°C in anaerobic conditions (AneroGen; Oxoid, Basingstoke, UK) for 3 days. The results are expressed as incidence of translocation in the event of positive growth on agar plates, even a single colony of any microorganism. Data of CFU/ g tissue are also given.

### Determination of total IgA and cytokine concentrations

IgA and IL-1β, IL6, IL10, TNF-α and IFN-γ cytokines were quantified in serum by Luminex assay using simplex kits for each immune parameter and ProcartaPlex Basic Mouse kits (eBioscence, Vienna, Austria). The parameters were measured in a Luminex 100 IS^™^ (Luminex Corporation, Madison, USA) in the Central Service of Support to Research [SCSIE], University of Valencia, Spain.

Cytokine concentrations were also quantified in jejunum samples using the Luminex assay. Samples of jejunum were homogenized in PBS buffer (pH 7.2) with protease inhibitors cocktail (Complete, Mini tablets, Roche life science, Mannheim Germany) and, after centrifugation (10.000 rpm, for 15 min at 4°C), the supernatant was used for cytokine determinations. These measurements were done in triplicate for each sample.

### Biochemical parameter analysis

Biochemical parameters were quantified in serum obtained by blood centrifugation (3000 rpm for 10 min at room temperature). The following enzymatic assay kits were used: alkaline phosphatase (ALP] Reagent, Fisher Diagnostics, Middletown, USA) and Alanine aminotransferase (ALT) and Amylase (BioVision Incorporated, Milpitas, USA).

Urine was collected before (time = 0) and after 6 days of treatment to determine urea and creatinine concentrations by enzymatic assay kits (Sigma-Aldrich, St.Louis, USA). Protein concentrations were measured by Bradford colorimetric assay (Bradford BIO-RAD, BIORAD, USA). These measurements were taken in triplicate for each sample.

### Histology and histometry

Sections of both the jejunum and colon were collected from each animal immediately after the sacrifice. Samples from five animals per group were immediately fixed in 4% para-formaldehyde in 0.01M phosphate-buffered saline (PBS) pH 7.4 for 24h at 4°C, dehydrated in a graded series of ethanol, cleared with xylene and embedded in paraffin. Serial microtome sections (3 μm-thick) were obtained from each sample and stained with haematoxylin/eosin (HE) to evaluate the structural aspects of both jejunum and colon, and for histometry. For histometry, the HE-stained sections were assessed to determine the goblet cells per intestinal villi, enterocyte height, height of intestinal villi (V) (10 villi measured per section), the depth of intestinal crypts (C) (10 crypts measured per section), and the villi and crypt ratio values. In addition, the gut associated lymphoid tissue (GALT) was examined, particularly the lymphoid area of individual follicles containing the Peyer’s patches on HE-stained colon sections. Measurements were taken from images obtained using a light microscope fitted with a NIKON, Olympus, Eclipse 90i, UK camera using NIS Elements BR 2.3 research software (Kingston; Surrey, KT2 5PR, England). Data are expressed as means and standard error of the mean. All intestinal tissue samples were examined for histological evidence of abnormality by an experienced histopathologist.

### RNA extraction and quantitative real time-PCR analysis

Total RNA from colon and liver was extracted with TRIzol reagent (Molecular Research Center, Inc.) and it was purified using the PureLink Total RNA Purification System (Invitrogen) following the manufacturer's instructions. All RNA samples inside the purification column were treated with RNase-Free DNase for removal of contaminating DNA (Invitrogen). Purified total RNA was stored at– 80°C until used as a template for cDNA synthesis. The total RNA was submitted to electrophoresis on 1% agarose gel to evaluate quality before quantification by spectrophotometry in a NanoDrop (Thermo Scientific Inc., Bremen, Germany). Only RNA samples with >200 μg/mL of RNA and A260/A280 ratio between 1.7 and 2.1 were analyzed. Total RNA was reverse transcribed in a 20 μL final volume from 300 ng/simple total RNA (DNA-free) using TaqMan Reverse Transcription Reagent kit (Applied Biosystems) according to the manufacturer's instructions. The following TaqMan Gene Expression Assays were purchased (Applied Biosystems, Barcelona, Spain): iNOS (Assay ID Mm00440502_m1), PPARγ (Assay ID Mm01184322-m1), TLR4 (Assay ID Mm00445273-m1), TLR2 (Assay ID Mm00442346-m1), NF-KB (Assay ID Mm00476361-m1), p38 (Assay ID Mm01301009-m1), CD14 (Assay ID Mm00438094-m1), Myd88 (Assay ID Mm0044338-m1) and β-actin (Assay ID Mm00607939_s1). Amplifications were carried out in a total volume of 20 μL containing 1x TaqMan Universal PCR Master Mix (Applied Biosystems). The amplification program was as follows: an initial cycle of 20 s at 95°C, followed by 40 cycles of 3 s at 95°C and 30 s at 60°C using the 7900 HT-Fast Real Time PCR System (Applied Biosystems). The relative differences in expression between groups were expressed using cycle threshold (Ct) values and the *ΔΔCt* method [18] as follows. The Ct values of the genes were first normalized in relation to β-actin in the same sample. Assuming that the Ct value is reflective of the initial starting copy and that there is 100% efficiency, a difference of one cycle is equivalent to a twofold difference in starting copy. Five replicas were analyzed per sample, and fold changes were generated for each sample by calculating 2-DDCT [19].

### Extraction of protein and Western blotting of PPAR-γ and iNOS

Frozen intestinal tissue samples (∼200 mg) were homogenized in ice cold RIPA buffer (1 x solution, 150 mM NaCl, 1.0% IGEPAL® CA-630, 0.5% sodium deoxycholate, 0.1% SDS, and 50 mM Tris, pH 8.0) (Sigma-Aldrich) and centrifuged at 10000 x g and 4°C for 10 min. Protein concentration was determined by the Bradford’s method using the commercial kit Bio-Rad protein assay (Bio-Rad laboratories)

Under denaturing conditions, samples (100 μg of proteins) were resolved by electrophoresis a 12% dodecylsulfate-polyacrylamide gel (SDS-PAGE) for PPAR-γ detection and in 8% SDS-PAGE for iNOS detection. The proteins were then transferred onto nitrocellulose Amersham Protran Western blotting membrane.

PPAR-γ was detected by a rabbit polyclonal antibody against amino acids 8–106 of PPAR-γ (sc-7196, Santa Cruz Biotechnology, Inc). iNOS was detected by a rabbit polyclonal antibody against a peptide mapping near the C-terminus of NOS2 (iNOS) of mouse origin (sc-650, Santa Cruz Biotechnology, Inc). The reaction was visualized by biotinylated secondary antibody followed by Amersham ECL select western blotting detection reagent. To control for protein degradation, the Western analysis was done in parallel for all samples using an antibody against β-actin (sc-130656, Santa Cruz Biotechnology, Inc).

### Statistical analysis

The results were analyzed using GraphPad Prism^®^ Version 5.0 (Graph Pad Software Inc., San Diego, CA, USA). All the results are expressed as the mean ± SEM. Two-way analysis of variance (ANOVA), and repeated measures ANOVA were applied for comparisons of means. Bonferroni test (when the variances were assumed to be equal) or Dunnett’s T3 test (when the variances were assumed to be unequal) was applied to perform *post hoc* pairwise multiple comparisons between groups. Differences between groups were established using an unpaired Student's *t*-test (data were normally distributed) or Mann-Whitney U test when the data were non-normally distributed. Significance was defined at P ≤ 0.05.

## Results

### General health, feed and water intake, and growth

Throughout the experimental period, there was no noticeable change in normal activity, behavior, or hair lustre in any of the groups of mice. There were no records of diarrhea or other treatment-related sickness or death. At the end of the experimental period, all animals were alive and healthy in all experimental groups.

There were no statistically significant difference in body weight gain or loss between immunocompetent and immunosuppressed animals treated or untreated with *B*. *uniformis* CET 7771 (Table 1).

**Table 1:** Body and tissue weight of control mice and immunosuppressed mice, fed either *B*. *uniformis* CECT 7771 or placebo.

Body and organs weight	Control	Control+B	IMM	IMM+B
Initial Body Weight (g)	20.98 ± 0.31	21.54 ± 0.29	21.44 ± 0.26	21.58 ± 0.30
Final Body Weight (g)	21.98 ± 0.40	22.10 ± 0.29	21.27 ± 0.36	20.80 ± 040
Weight increment (g)	1.07 ± 0.39	0.56 ± 0.23	(-) 0.17 ± 0.75	(-) 0.62 ± 1.33
MLNs (mg)	33.7 ± 0.4	41.8 ± 0.4	35.3 ± 0.3	34.9 ± 0.3
Liver (mg)	975.5 ± 65.3	980.1 ± 120.3	974.3 ± 180.2	983.9 ± 78.3

Control, mouse group fed placebo daily by gavage for 6 days (n = 10); Control+B, mouse group that received a daily dose of 2 x10^9^ CFU *B*. *uniformis* CECT 7771 by gavage for 6 days (n = 10); IMM, immunosuppressed mouse group fed placebo daily by gavage for 6 days (n = 10); and IMM+B, immunosuppressed mouse group receiving a daily dose of 2 x10^9^CFU *B*. *uniformis* CECT 7771 by gavage for 6 days (n = 10).

MLNs mesenteric lymph nodes

Data are expressed as mean values with SEM, n = 10. Differences between groups were established using an unpaired Student's t-test at p<0.05.

Concerning tissue weight, there were no significant differences in the weight of liver and MLN between controls and mice orally administered *B*. *uniformis* CET 7771 (Table 1). The coloration and appearance of the analyzed tissues was normal, and no differences were observed between groups.

### Bacterial Translocation

The incidence of translocation of bacteria from the gut to different tissues is shown in Table 2 No bacteremia was observed in any of the experimental groups. Although some events of bacterial translocation to liver and MLN were recorded, there was no statistically significant difference in the incidence of these events between control and treated groups, suggesting that they were due to cross-contamination during animal dissection.

**Table 2:** Bacterial translocation in MLNs, liver and blood of immunocompetent and immunosuppressed mice, fed either *B*. *uniformis* CECT 7771 or placebo.

Body organs	Control (n = 10)		Control + B (n = 10)		IMM (n = 10)		IMM + B (n = 10)	
Culture media								
	SCHA	WILK	SCHA	WILK	SCHA	WILK	SCHA	WILK
CFU/g* (n° positive samples per group when appropriate)								
MLNs	0	0	0	0	0	0	0	0
Liver	3 (5/10)	>50 (10/10)	1 (4/10)	>50 (10/10)	1 (5/10)	>50 (10/10)	0 (10/10)	>50 (9/10)
Blood	0	0	0	0	0	0	0	0

Control; Control + B receiving a daily dose of 2 x10^9^ CFU *B*. *uniformis* CECT 7771 by gavage for 6 days; IMM, immunosuppressed mice and IMM + B, immunosuppressed mice group receiving a daily dose of 2 x10^9^CFU *B*. *uniformis* CECT 7771 by gavage for 6 days.

MLNs mesenteric lymph nodes

SCHA = Schaedler anaerobe agar, specifically for *Bacteroides* spp.

WILK = Wilkins–Chalgren agar, for strict anaerobic bacteria

*Data are expressed as colony forming unit (cfu) per gram of tissue.

The number of positive organs and the number of cfu found is consistent with studies published previously, where events of cross-contamination eventually lead to detect bacteria in diverse organs from control animals [5, 20].

### Biochemical parameters

**Fig 1:**
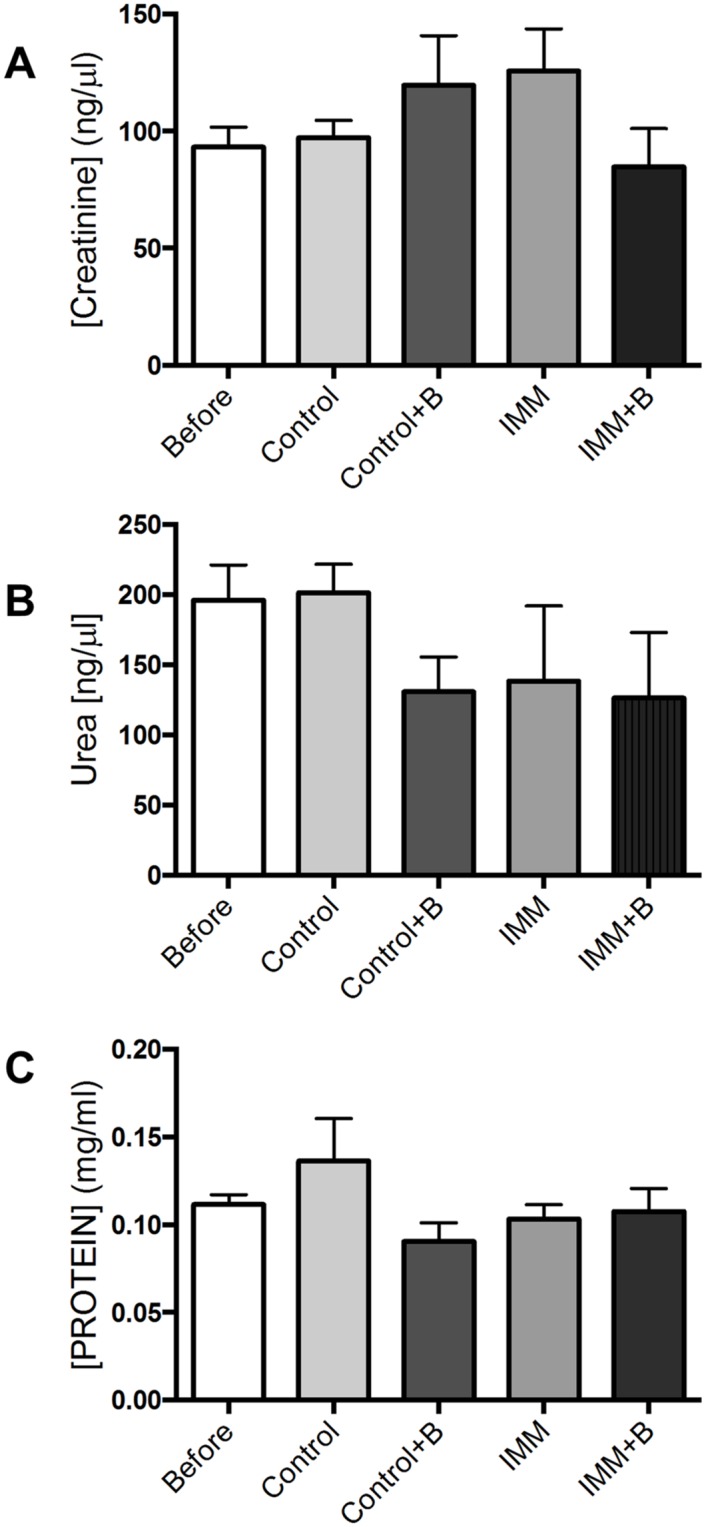
Urine biochemistry measurements in immunocompetent and immunosuppressed mice, fed either *B*. *uniformis* CECT 7771 or placebo. Control, mouse group fed placebo daily by gavage for 6 days (n = 10); Control + B, mouse group that received a daily dose of 2 x10^9^ CFU *B*. *uniformis* CECT 7771 by gavage for 6 days (n = 10); IMM, immunosuppressed mouse group fed placebo daily by gavage for 6 days (n = 10); and IMM+B, immunosuppressed mouse group receiving a daily dose of 2 x10^9^CFU *B*. *uniformis* CECT 7771 by gavage for 6 days (n = 10). Data are expressed as means and SEM. The differences were determined by applying the Mann-Whitney U test. In every case, p-values <0.05 were considered statistically significant. Measurements were taken in triplicate for each sample.

**Fig 2:**
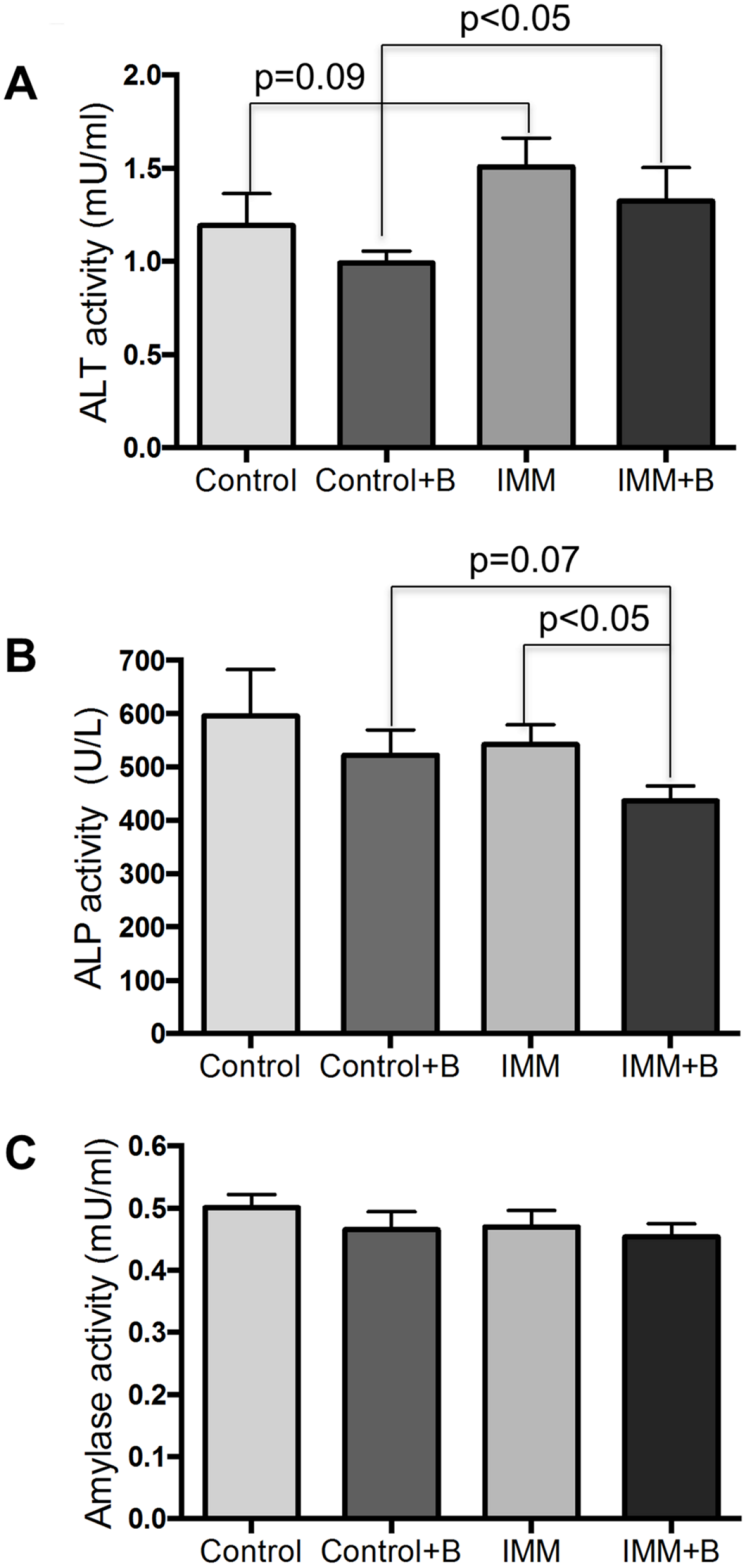
Blood biochemistry measurements in immunocompetent and immunosuppressed mice, fed either *B*. *uniformis* CECT 7771 or placebo. Control, mouse group fed placebo daily by gavage for 6 days (n = 10); Control + B, mouse group that received a daily dose of 2 x10^9^ CFU *B*. *uniformis* CECT 7771 by gavage for 6 days (n = 10); IMM, immunosuppressed mouse group fed placebo daily by gavage for 6 days (n = 10); and IMM+B, immunosuppressed mouse group receiving a daily dose of 2 x10^9^CFU *B*. *uniformis* CECT 7771 by gavage for 6 days (n = 10). Data are expressed as means and SEM. The differences were determined by applying the Mann-Whitney U test. In every case, p-values <0.05 were considered statistically significant. Measurements were taken in triplicate for each sample.

In order to detect signs of possible lesions in different organs due to oral bacteroides administration, biochemical parameters related to pancreatic, liver and kidney functions were analyzed (Figs 1 and 2). Oral administration of *B*. *uniformis* CECT 7771 did not cause significant changes in urine parameters (Fig 1) nor in serum amylase activity (Fig 2C) between the different mice groups studied. The immunosuppressed treatment induced an increase in ALT activity of serum, independently of bacteroide administration, although the effect was more remarkable in the groups receiving the bacteroide due to lower serum levels of the Control+B group (Fig 2A). The ALP activity was significantly reduced in the bacteroide-fed immunosuppressed mice compared to placebo-fed immunosuppressed group (p<0.05). Moreover, when comparing immunocompetent and immunosuppressed mice we observed that the immunosuppression induced a decrease in ALP activity of serum (p = 0.07).

### Jejunum and colon mucosal histology effects

**Fig 3:**
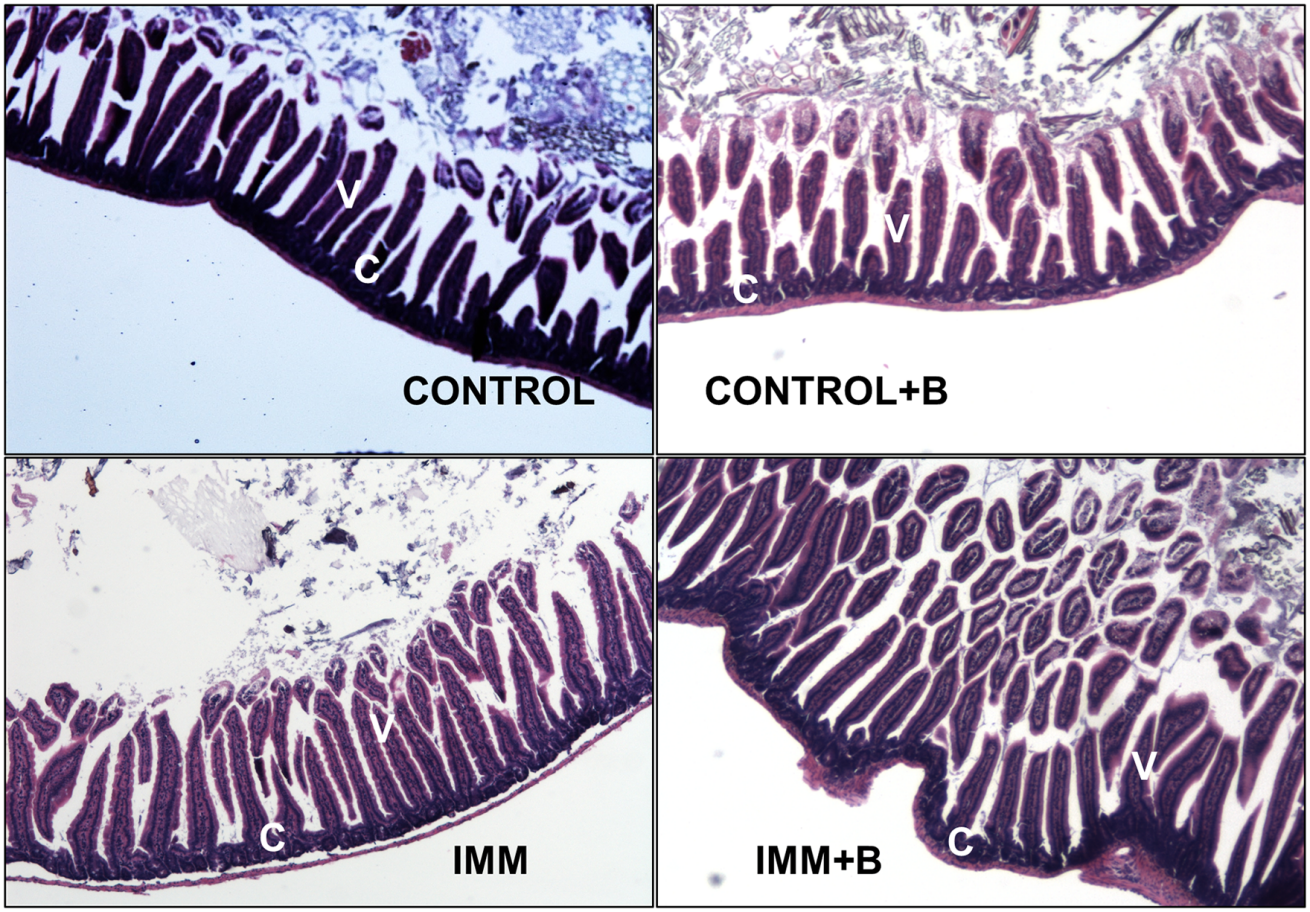
Histology of jejunum sections from immunocompetent and immunosuppressed mice, fed either *B*. *uniformis* CECT 7771 or placebo. Control, mouse group fed placebo daily by gavage for 6 days (n = 5); Control + B, mouse group that received a daily dose of 2 x10^9^ CFU *B*. *uniformis* CECT 7771 by gavage for 6 days (n = 5); IMM, immunosuppressed mouse group fed placebo daily by gavage for 6 days (n = 5); and IMM+B, immunosuppressed mouse group receiving a daily dose of 2 x10^9^CFU *B*. *uniformis* CECT 7771 by gavage for 6 days (n = 5). Data are expressed as means and SEM. The differences were determined by applying the Mann-Whitney U test. In every case, p-values <0.05 were considered statistically significant. These measurements were taken in 10 fields for each sample. Photomicrographs 4x of representative HE-stained slides are shown. V = Villi and C = crypt.

**Fig 4:**
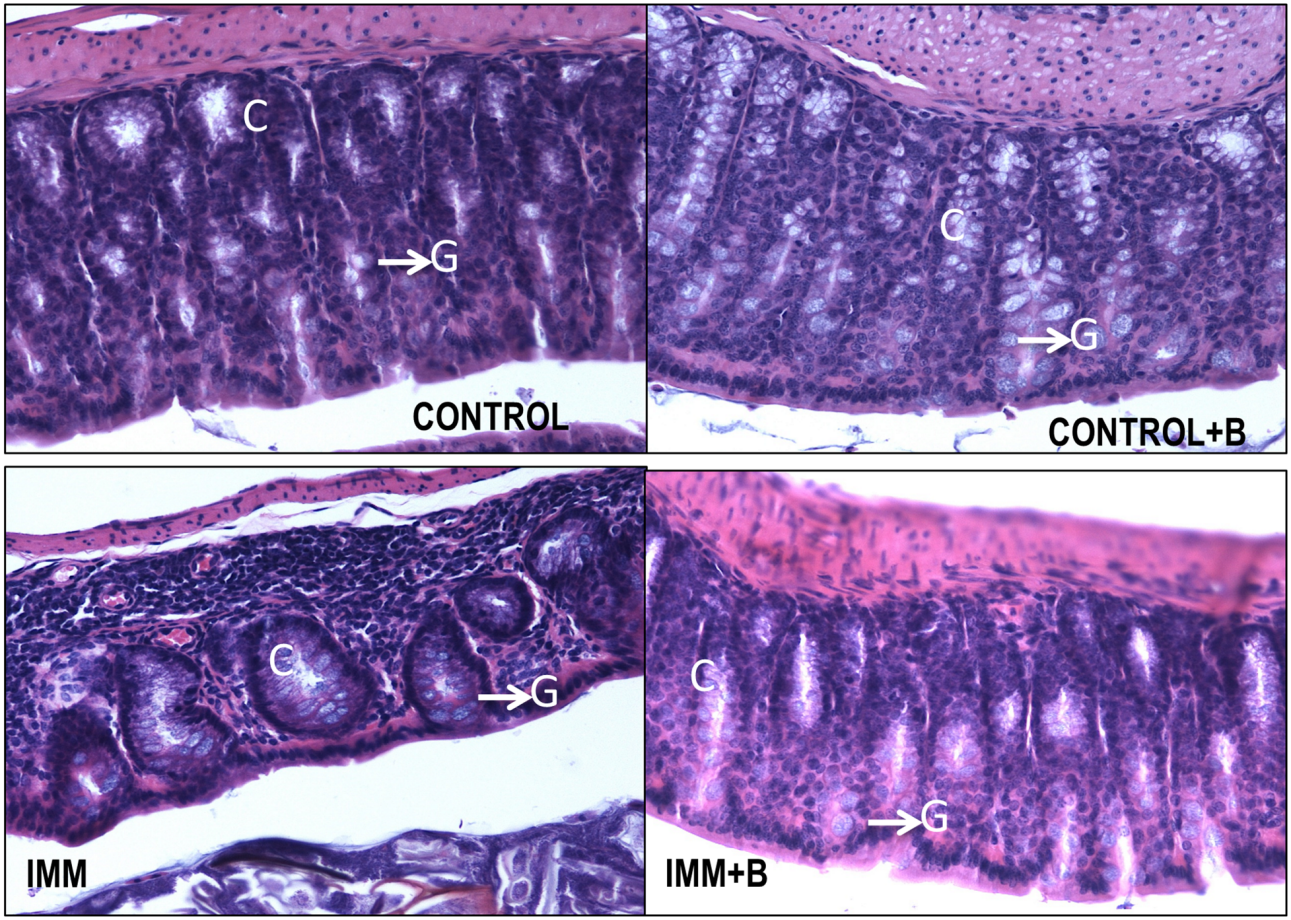
Histology of colon section from immunocompetent and immunosuppressed mice, fed either *B*. *uniformis* CECT 7771 or placebo. Control, mouse group fed placebo daily by gavage for 6 days (n = 5); Control + B, mouse group that received a daily dose of 2 x10^9^ CFU *B*. *uniformis* CECT 7771 by gavage for 6 days (n = 5); IMM, immunosuppressed mouse group fed placebo daily by gavage for 6 days (n = 5); and IMM+B, immunosuppressed mouse group receiving a daily dose of 2 x10^9^CFU *B*. *uniformis* CECT 7771 by gavage for 6 days (n = 5). Data are expressed as means and SEM. The differences were determined by applying the Mann-Whitney *U* test. In every case, p-values <0.05 were considered statistically significant. These measurements were taken in 10 fields for each sample. Photomicrographs 20 x of representative HE-stained slides are shown. G, goblet cells; C, crypts.

The effect of the intervention on jejunum and colon histology is shown in Table 3 and Figs 3 and 4, respectively. In jejunum, immunocompetent and immunosuppressed group showed slight, but not significant, differences in some of the parameters measured, except for villus height/crypt depth ratio, which was slightly reduced (p = 0.06) and in the number of goblet cells/villus ratio (p<0.01) in immunosuppressed mice (Table 3 and Fig 3). However, the immunosuppressed mice treated with the bacteroide showed values of crypts depth and villus height/crypt depth in the jejunum similar to immunocompetent mice, restoring the alterations caused by immunosuppression. The Lieberkühn crypts had a uniform and constant appearance (Fig 3). The immunosuppressed mice also showed a significant decrease in crypt depth in colon compared to control mice; however, effects of bacteroides administration were undetectable (Table 3). Control mice treated with *B*. *uniformis* CECT 7771 exhibited a significant increase in goblet cells/villus ratio in the colon (p< 0.01) (Table 3, Fig 4, IMM+B) compared to mice fed placebo.

**Table 3:** Gut mucosal histology measurements of immunocompetent and immunosuppressed mice, fed *B*. *uniformis* CECT 7771 or placebo.

	Control (n = 5)	Control + B (n = 5)		IMM (n = 5)		IMM + B (n = 5)	
**Jejunum**							
			p^a^		p^b^		p^c^
Villus height (μm)	290.21 ± 18.74	278.21±15.64	>0.05	246.10±23.27	>0.05	253.3± 18.03	> 0.05
Crypt depth (μm)	58.39 ±9.23	65.48 ± 2.46	>0.05	67.76±3.55	>0.05	57.04 ± 2.24	< 0.05
Villus width (μm)	37.42 ± 3.23	35.47 ± 2.24	>0.05	45.80 ±5.63	>0.05	38.54 ± 2.53	>0.05
Villus height/crypt depth ratio	4.50 ± 0.44	4.49 ± 0.36	>0.05	3.71 ± 0.34	= 0.06	4.70 ± 0.46	= 0.06
Number of goblet cells/villus	11 (10–12)	8 (6–11)	>0.05	4 (3–5)	<0.01	4 (3–5)	>0.05
Enterocyte height (μm)	12.95± 0.77	13.48±0.89	>0.05	12.41±0.58	>0.05	11.45±0.59	>0.05
Enterocyte:lymphocyte ratio	4:1	5:1	>0.05	4:1	>0.05	5:1	>0.05
**Colon**							
			p^a^		p^b^		p^c^
Crypt depth (μm)	94.98± 46.97	91.91 ± 38.44	>0.05	64.57 ± 18.54	= 0.012	76.88 ± 29.57	>0.05
Number of goblet cells/villus	7 (6–14)	16 (13–18)	< 0.01	8 (5–16)	>0.05	10 (5–11)	>0.05

Control, mouse group that received placebo by gavage for 6 days; Control+B, group that received a daily dose of 2 x10^19^ CFU *B*. *uniformis* CECT 7771 by gavage for 6 days; IMM, immunosuppressed mouse group that received placebo by gavage for 6 days and IMM+B, immunosuppressed mouse group that received a daily dose of 2x10^9^ CFU *B*. *uniformis* CECT 7771 by gavage for 6 days.

^a^ Control vs Control+B,

^b^ Control vs IMM

^c^ IMM vs IMM+B

Data are expressed as mean values with SEM, n = 5 samplesx10measures. Significant differences were established at p<0.05 by using t-student test.

There were no statistically significant differences in number and size of lymphatic follicles between immunocompetent and immunosuppressed mice, nor was lymphocytic infiltrate apparent (Figs 3 and 4).

### Immunological effects

**Fig 5:**
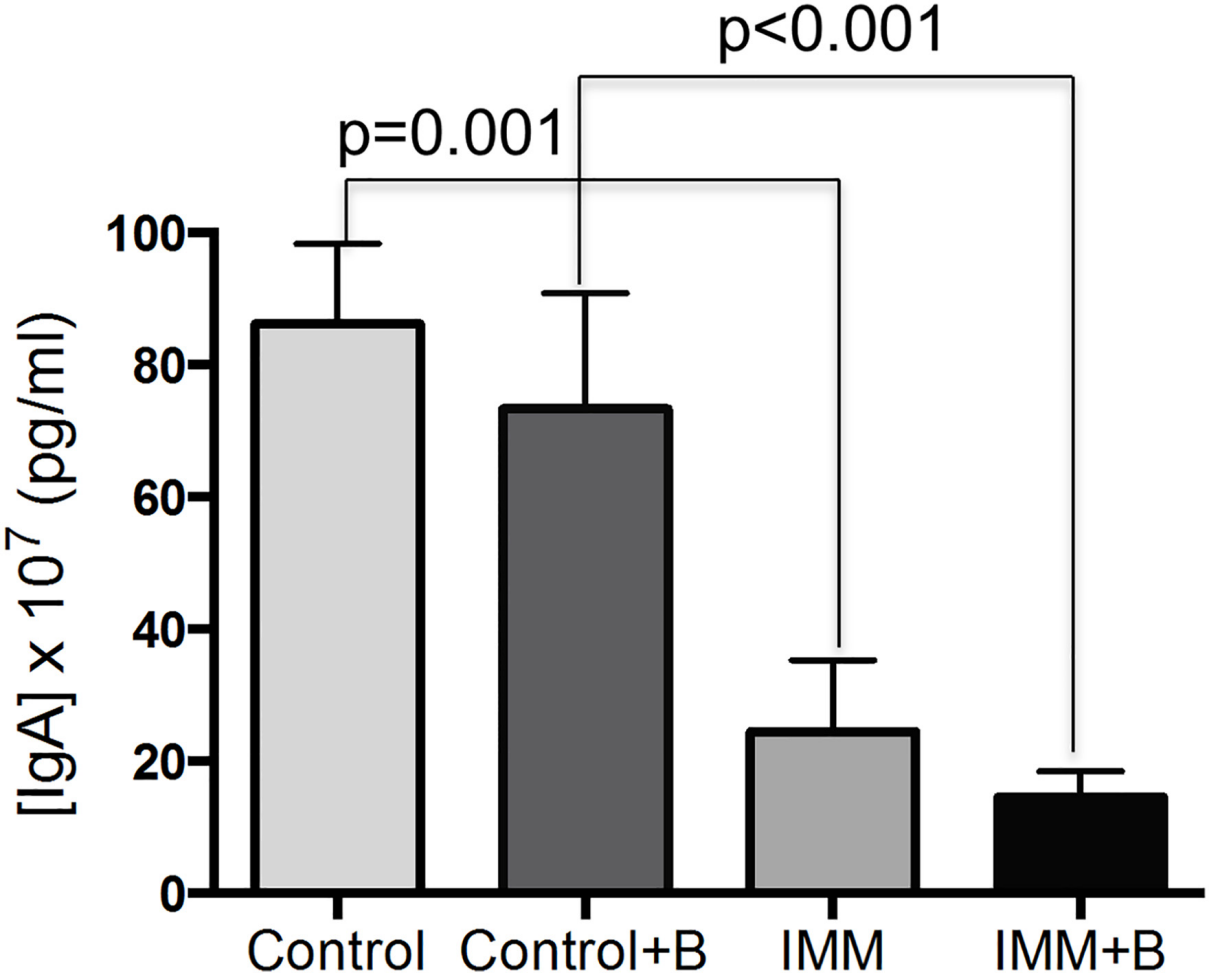
The measurement of serum IgA concentration in immunocompetent and immunosuppressed mice, fed either *B*. *uniformis* CECT 7771 or placebo. Control, mouse group fed placebo daily by gavage for 6 days (n = 10); Control + B, mouse group that received a daily dose of 2 x10^9^ CFU *B*. *uniformis* CECT 7771 by gavage for 6 days (n = 10); IMM, immunosuppressed mouse group fed placebo daily by gavage for 6 days (n = 10); and IMM+B, immunosuppressed mouse group receiving a daily dose of 2 x10^9^CFU *B*. *uniformis* CECT 7771 by gavage for 6 days (n = 10). Data are expressed as means ± SEM. The differences were determined by applying the Mann-Whitney *U* test. In every case, p-values <0.05 were considered statistically significant. These measurements were taken in triplicate for each sample.

**Fig 6:**
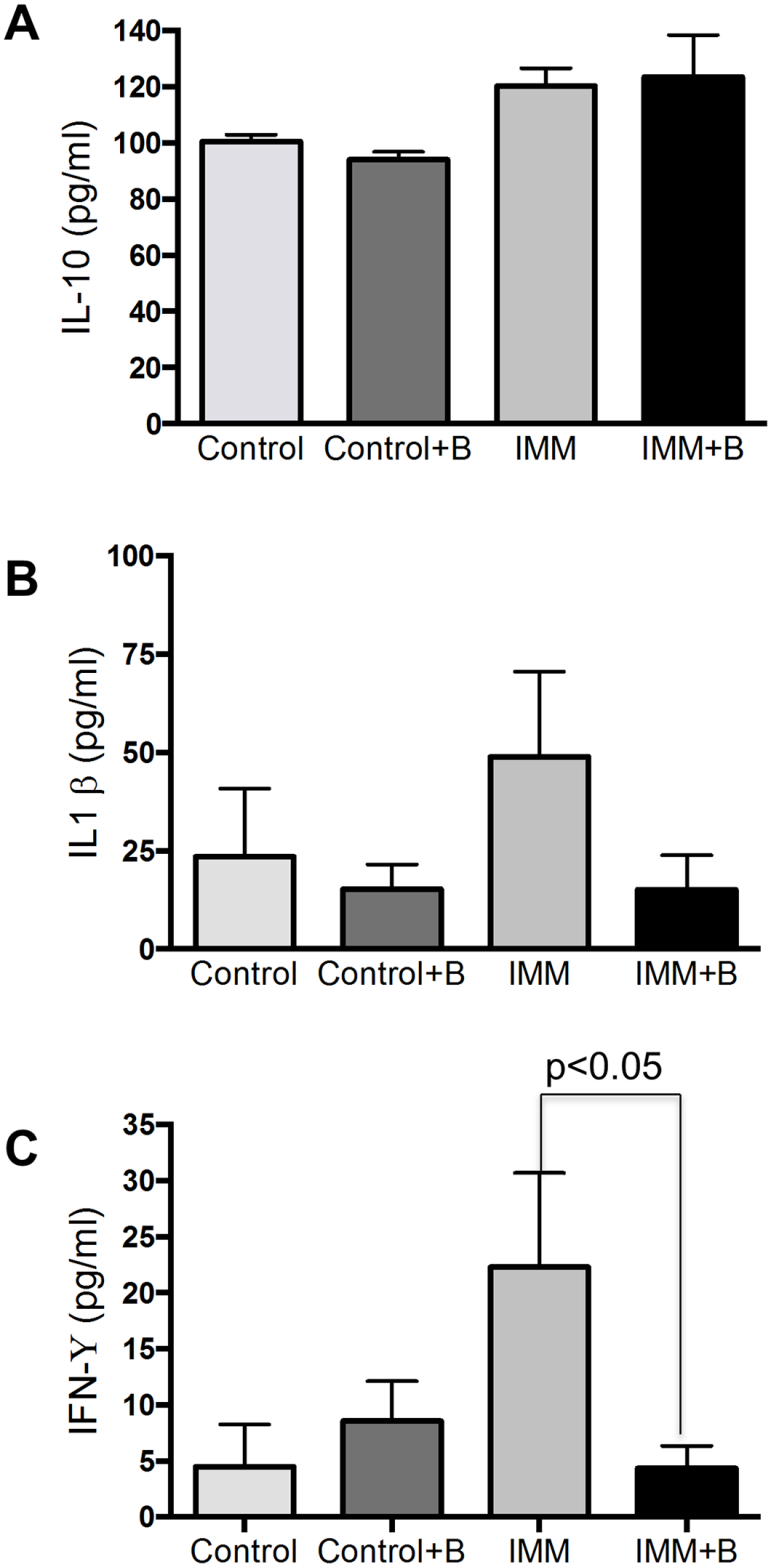
Cytokine production in jejunum samples from immunocompetent and immunosuppressed mice, fed either *B*.*uniformis* CECT 7771 or placebo. Control, mouse group fed placebo daily by gavage for 6 days (n = 10); Control + B, mouse group that received a daily dose of 2 x10^9^ CFU *B*. *uniformis* CECT 7771 by gavage for 6 days (n = 10); IMM, immunosuppressed mouse group fed placebo daily by gavage for 6 days (n = 10); and IMM+B, immunosuppressed mouse group receiving a daily dose of 2 x10^9^CFU *B*. *uniformis* CECT 7771 by gavage for 6 days (n = 10). Data are expressed as means ± SEM. The differences were determined by applying the Mann-Whitney *U* test. In every case, p-values <0.05 were considered statistically significant. These measurements were taken in triplicate for each sample.

As expected, the serum IgA concentration was higher (p = 0.001) in the immunocompetent mouse groups than in immunosuppressed ones (Fig 5). The concentrations of the cytokines assayed (IL-1β, IL10, IFNγ IL6 and TNF-α) were below the detection limit in serum (data not shown). In jejunum samples IL-1β, IL10 and IFN-γ concentrations were detected (Fig 6), but no statistical differences were found between the four experimental groups for IL-1β, and IL10 (Fig 6A and 6B). In contrast, immunosuppressed mice had higher IFN-γ concentrations than immunosuppressed mice fed *B*. *uniformis* CECT 7771 in jejunum samples (Fig 6C).

### Gene expression effects in colon and liver

**Fig 7:**
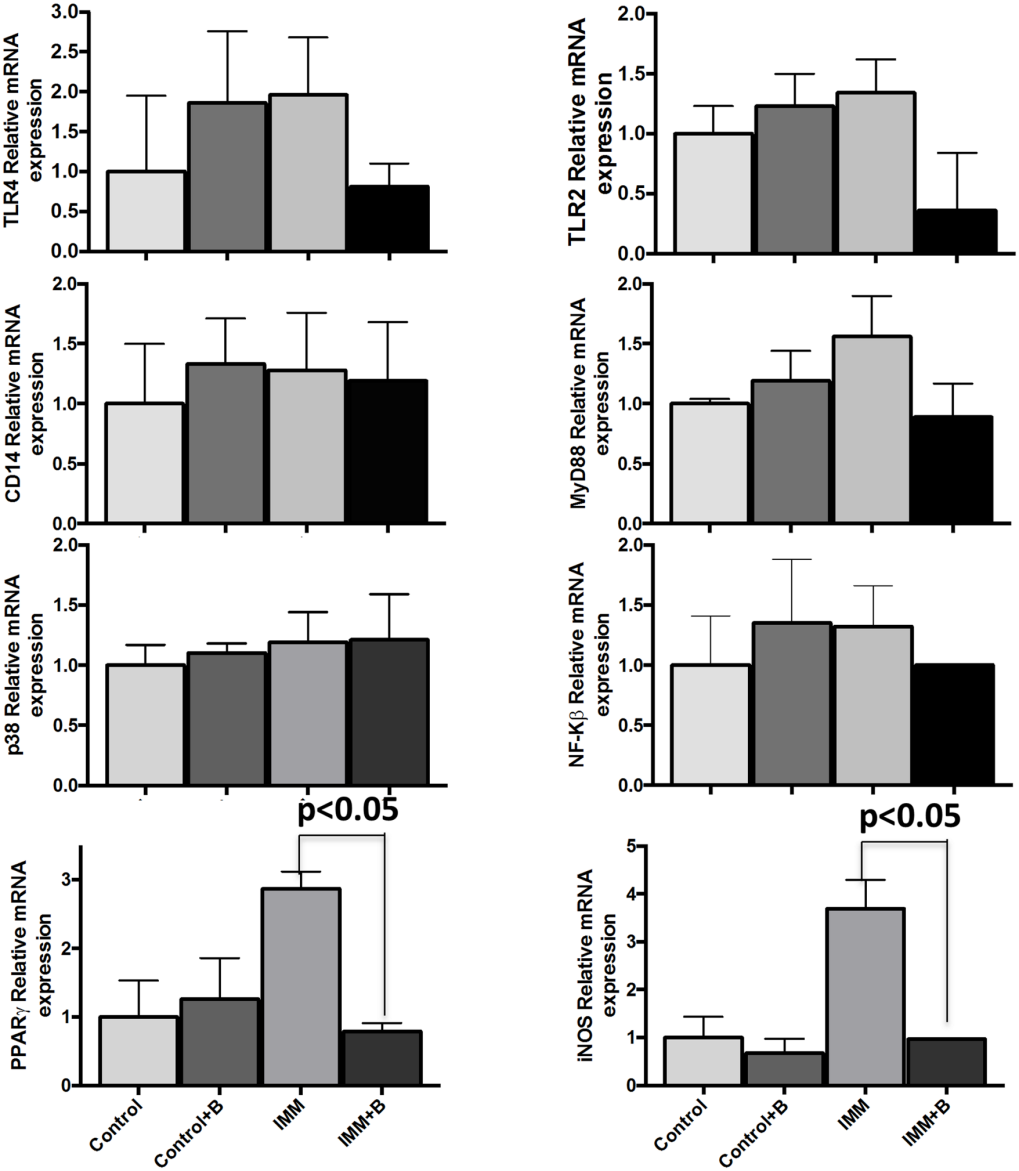
Gene expression assays in colon from immunocompetent and immunosuppressed mice, fed either *B*.*uniformis* CECT 7771 or placebo. Control, mouse group fed placebo daily by gavage for 6 days (n = 5); Control + B, mouse group that received a daily dose of 2 x10^9^ CFU *B*. *uniformis* CECT 7771 by gavage for 6 days (n = 5); IMM, immunosuppressed mouse group fed placebo daily by gavage for 6 days (n = 5); and IMM+B, immunosuppressed mouse group receiving a daily dose of 2 x 10^9^CFU *B*. *uniformis* CECT 7771 by gavage for 6 days (n = 5). Data are expressed as the mean value ± SEM. Differences between groups were established using an unpaired Student's *t*-test. Results with a two-sided *p*-value <0.05 were considered statistically significant. These measurements were taken in triplicate for each sample.

**Fig 8:**
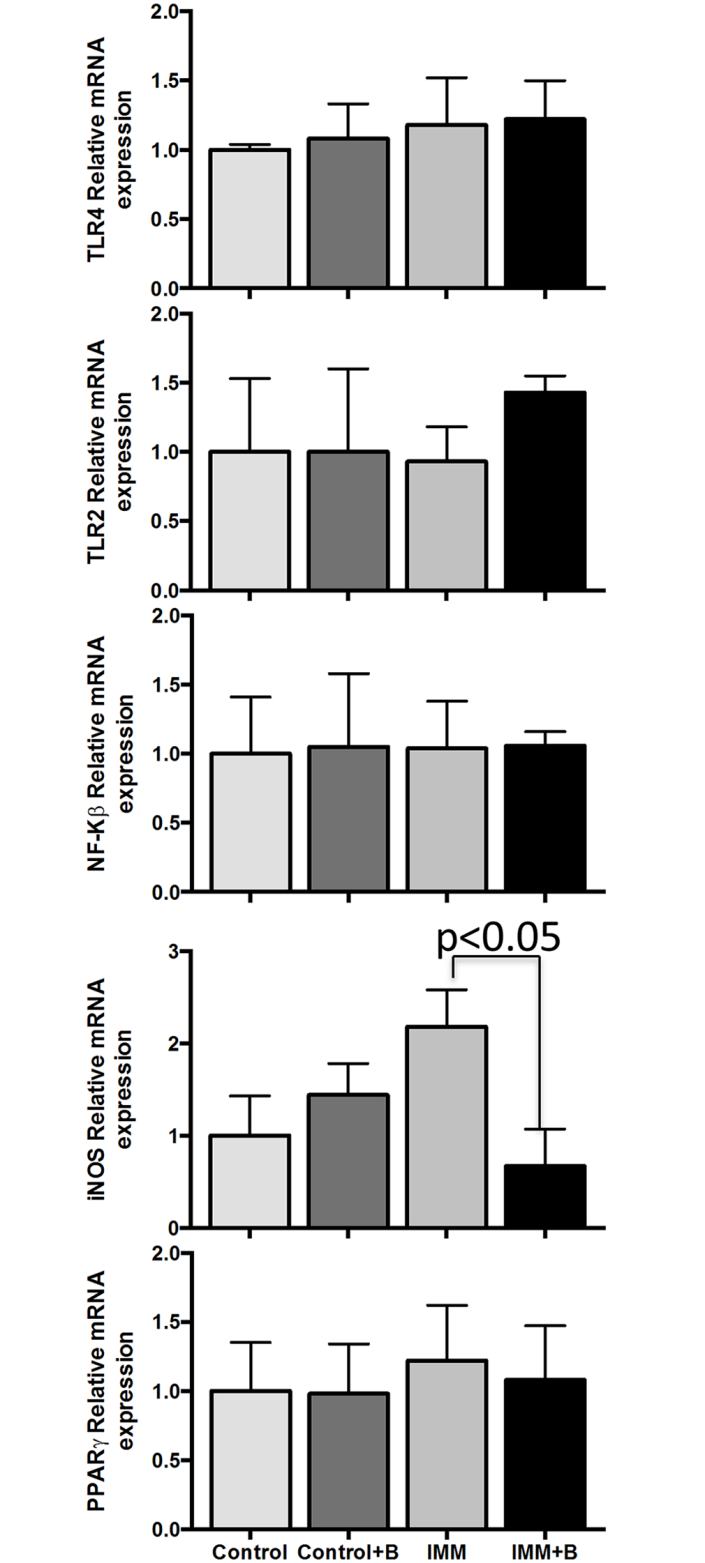
Liver gene expression assays in immunocompetent and immunosuppressed mice, fed either *B*.*uniformis* CECT 7771 or placebo. Control, mouse group fed placebo daily by gavage for 6 days (n = 5); Control + B, mouse group that received a daily dose of 2 x 10^9^ CFU *B*. *uniformis* CECT 7771 by gavage for 6 days (n = 5); IMM, immunosuppressed mouse group fed placebo daily by gavage for 6 days (n = 5); and IMM+B, immunosuppressed mouse group receiving a daily dose of 2 x10^9^CFU *B*. *uniformis* CECT 7771 by gavage for 6 days (n = 5). Data are expressed as the mean value ± SEM. Differences between groups were established using an unpaired Student's *t*-test. Results with a two-sided *p*-value <0.05 were considered statistically significant. These measurements were taken in triplicate for each sample.

Gene expression comparison between immunosuppressed mice and immunocompetent mice fed *B*. *uniformis* CECT 7771 (IMM+B) or placebo (IMM) are shown in Figs 7 and 8.

Gene expression of components of the TLR4 and TLR2 pathways (TLR4, TLR2, CD14, MyD88, p38 and NF-Κβ genes) did not exhibit significant changes between the four mouse groups in any of the tissue studied (Figs 7 and 8).

Only *PPARγ* and *iNOS* mRNA expression was upregulated in colon and liver of immunosuppressed mice compared to control mice. *Bacteroides* administration downregulated the increased *iNOS* and *PPARγ* gene expression detected in immunosuppressed mice (Fig 7). Liver immunosuppressed mice also showed increased *iNOS* expression (p<0.05) and this effect was reversed by the *Bacteroides* strain tested (Fig 8).

### PPARγ and iNOS protein expression in colon

**Fig 9:**
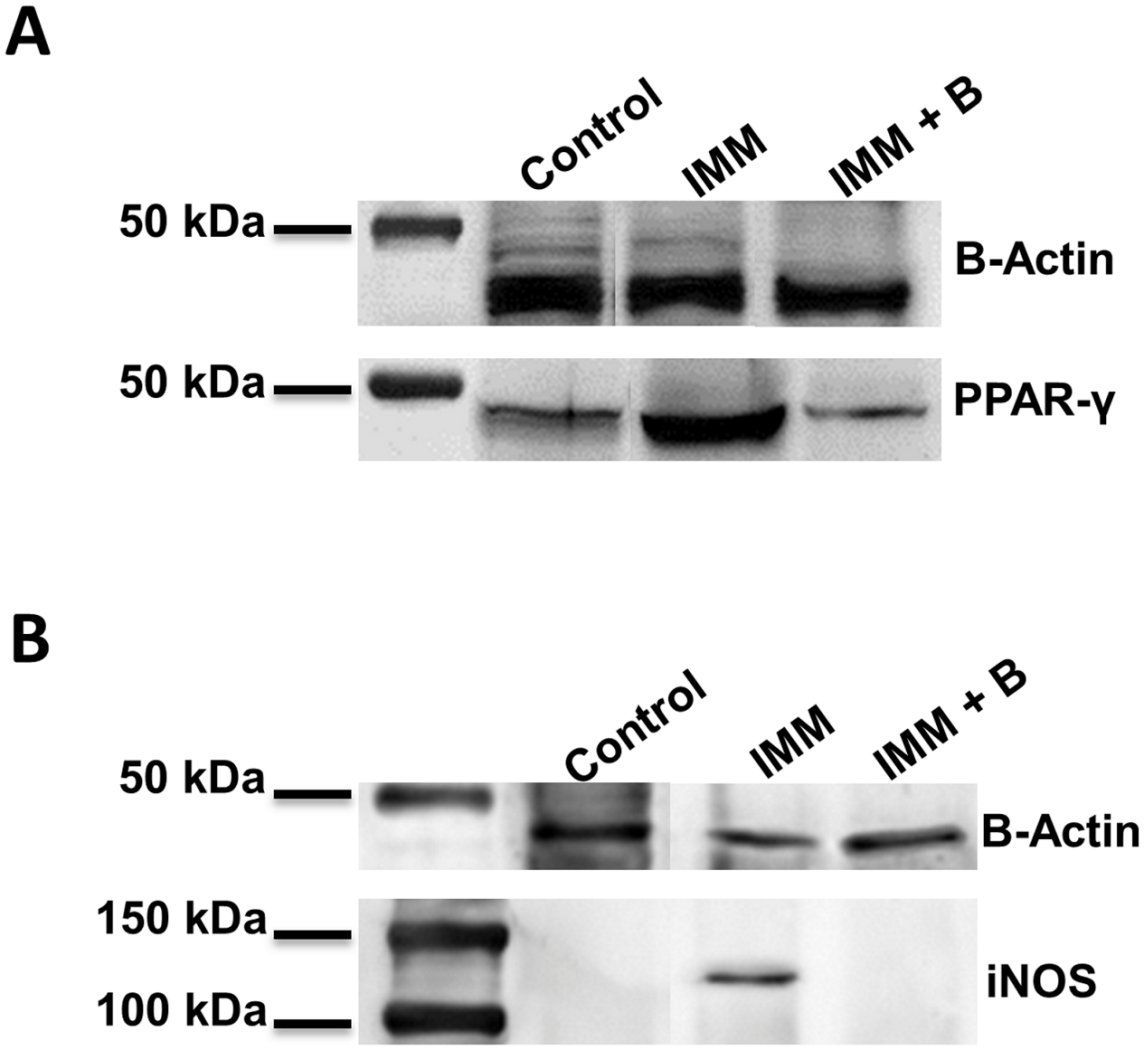
PPAR-γ and iNOS protein expression in colon from immunocompetent and immunosuppressed mice, fed either *B*.*uniformis* CECT 7771 or placebo. (A) PPAR-γ and (B) iNOS protein expression from colon protein samples were visualized using western blot analysis. Control, mouse group fed placebo daily by gavage for 6 days (n = 5); Control + B, mouse group that received a daily dose of 2 x10^9^ CFU *B*. *uniformis* CECT 7771 by gavage for 6 days (n = 5); IMM, immunosuppressed mouse group fed placebo daily by gavage for 6 days (n = 5); and IMM+B, immunosuppressed mouse group receiving a daily dose of 2 x10^9^CFU *B*. *uniformis* CECT 7771 by gavage for 6 days (n = 5).

As shown in the Western blot of colon fractions (Fig 9), immunosuppression treatment increased PPARγ and iNOS protein expression in mice compared to controls. In contrast, the *Bacteroides* administration ameliorated those effects on PPARγ and iNOS protein expression detected in immunosuppressed mice (Fig 9A and 9B, respectively). This analysis confirmed results obtained by gene expression analysis (Fig 7)

## Discussion

The majority of commercialized probiotics (classical probiotics) are still restricted to lactic acid bacteria, such as the genera *Lactobacillus*, as well as to bifidobacteria, isolated from biological samples and often derived from fermented foods owing to their safety status based on their long history of consumption in traditional fermented products without causing concerns [21]. However, other indigenous species and strains that are dominant inhabitants of the human intestine could constitute a next generation of probiotics with improved efficacy [1]. In fact, 80–90% of bacterial phylotypes are members of two phyla: Bacteroidetes (including the genera *Bacteroides* and *Prevotella*) and Firmicutes [22]. Relative proportions of Firmicutes and Bacteroides have been related to the host metabolic phenotype (e.g. lean and obese). Within this phylum, the genus *Bacteroides* or subgroups have also been associated with a lean phenotype or weight loss in observational human studies, although contradictions exist in the literature [23]. Breast-feeding also seems to increase the abundance of *B*. *uniformis* in the fecal microbiota of healthy infants [1, 16], as compared to formula-feeding; furthermore, breast-feeding reduces the risk of developing obesity and type-2 diabetes [24]. Therefore, a strain of this species, *B*.*uniformis* CECT 7771, was selected among other human infant *Bacteroides* strains for its anti-inflammatory potential *in vitro* and evaluated in mice with high-fat-diet induced obesity [3]. The administration of *B*. *uniformis* CECT 7771 ameliorated diet-induced metabolic and immune dysfunction associated with intestinal dysbiosis in obese mice. In the light of this evidence, and given the potential use of this strain for obesity management in humans, here we evaluated its safety by monitoring acute daily intake in immunosuppressed and immunocompetent mice.

There are no general guide-lines for safety evaluation of new strains to be considered as potential probiotics; however, rodent studies of acute oral toxicity have been proposed as a basic test for the assessment of probiotic safety [25] and applied previously in safety assessment studies [5,20–26]. The oral toxicity assessment showed that mice treated with a high dose of the potential probiotic strain *B*. *uniformis* CECT 7771 were healthy after daily oral administration for 6 days. This relative dose is 100 times higher than in humans when normalized by body surface area. No adverse effects were observed on body weight, food intake or other general indicators of the animals’ health status as a consequence of *B*. *uniformis* CECT 7771 administration. Similar results were obtained in immunosuppressed mice with the classical probiotc strains of the species *Lactobacillus rhamnosus*, *Bifidobacterium longum* and *Bifidobacterium breve* [27].

Translocation of bacteria from the intestinal lumen to MLN and other tissues may constitute a risk for systemic infection (bacteremia). Therefore, we assessed translocation of bacterium studied as an indication of potential infectivity and pathogenicity [28]. Although most *Bacteroides* spp. are considered commensals some strains of *B*. *fragilis* are opportunistic pathogens and have been involved in bacteraemia, which also justify to assess translocation of other bacteroides in a first safety assessment [29]. In spite of the high dose of *B*. *uniformis* CECT 7771 used for the feeding trial, no translocation of bacteria to MLN or blood was detected in the bacteroide-treated groups. Although bacterial counts were eventually found in liver, translocation events were similar in mice receiving the potential probiotic and control mice. This fact suggests that translocation was not associated with treatment but with cross-contamination. The presence of some bacteria in the liver has been described in healthy mice in previous safety studies [20, 30].

Biochemical parameters were determined in urine and blood to detect potential adverse sub-clinical effects of the strain tested. Urea, creatinine and protein concentration are routine markers of kidney function, low levels of urea or creatinine and high levels of protein in urine may indicate renal failure. The urine parameters were similar in all four mouse groups, suggesting the absence of adverse effects on key organs due to bacteroides administration. ALT and ALP are indicators of liver function, and amylase of the pancreatic function. We observed that ALT activity increased slightly in both immunosuppressed groups independently of oral administration of the bacteroide. Amylase activity was not affected by the either immunosuppression or bacteroides administration. The liver is the main organ responsible for the removal of lipopolysaccharide (LPS) coming from the gut from the circulation, which may have adverse health consequences. In this context, ALP plays an important role by dephosphorylating LPS which induces a 100-fold reduction in lipid A toxicity [31]. In our study, the ALP activity showed a slight decrease in immunosuppressed mice treated with *B*. *uniformis* CECT 7771 compared to immunosuppressed mice fed placebo, which would suggest that the bacteroides reduces endotoxemia in immunocompromised mice although differences were not statistically significant to draw conclusions in this regard.

The intestinal mucosa has an important barrier function, preventing potential pathogens and toxigenic substances from invading other tissues systemically [5, 30]. In this study, oral administration of *B*. *uniformis* CECT 7771 did not reveal any adverse effects on the integrity of the gut mucosa. However, immunosuppression caused a statistically significant decrease in the number of goblet cells in the jejunum and in crypt width in the colon, and a slight reduction in villus height/crypt depth ratio in the jejunum compared to controls. By contrast, the administration of the bacteroides to immunosuppressed mice reversed the reduced villus height/crypt depth ratio; moreover, the other alterations seen in immunosuppressed mice were unaffected. Administration of *B*. *uniformis* CECT 7771 also increased the number of goblet cells in the colon, which could enhance the production of the mucus coating antigens and pathogens and constituting a first line of defense before immune system activation. Similar observations have been made when evaluating strains of the genera *Bifidobacterium* and *Propionibacterium* [32], such as *Bifidobacterium longum* [5].

As specified above, the selection of *B*. *uniformis* CECT 7771 was based on previous *in vitro* studies demonstrating its ability to induce high levels of anti-inflammatory cytokines, together with low levels of pro-inflammatory cytokines after stimulation of Raw264.7 macrophages cultures [3]. Also positive effects have been detected *in vivo* in mice with and without diet-induced obesity [3]. The present study evaluated the effects of the same strain on a normal and suppressed immune system to provide further evidence of safety in conditions of increased risk of translocation and immune reaction. Immunosuppression was evident from the reduced IgA concentrations in blood, which was not reversed by the bacteroides administration. *B*. *uniformis* CECT 7771 reduced concentrations of pro-inflammatory cytokines (mainly IFN-γ and slightly IL1β) in the jejunum of immunosuppressed mice, which may reduce the development of inflammatory overreaction in immunocompromised subjects. We also studied the effect of *B*. *uniformis* CECT 7771 on the expression of genes directly involved in bacterial recognition. TLR pathways play a critical role in the early innate immune response to invading microorganisms or commensals [33]. TLRs recognize highly conserved structural motifs known as pathogen-associated microbial patterns (PAMPs) and danger-associated molecular patterns (DAMPs), which are endogenous molecules released from necrotic or dying cells. Stimulation of TLRs by the corresponding PAMPs or DAMPs initiates signaling cascades, leading to the activation of transcription factors, such as AP-1, NF-κβ and interferon regulatory factors (IRFs) [33]. TLR signaling results in a variety of cellular responses, including the production of interferons (IFNs), pro-inflammatory cytokines and effector cytokines, which direct the adaptive immune response. TLR2 is essential for the recognition of a variety of PAMPs from Gram-positive bacteria, including bacterial lipoproteins, lipomannans and lipoteichoic acids, while TLR4 is predominantly activated by lipopolysaccharide (LPS) of Gram-negative bacteria [33, 34]. In particular, epithelial TLR2 activation can protect against barrier disruption [34]. In contrast, activation of TLR 4 and NF-Kβ usually lead to an inflammatory response that could also increase intestinal permeability and enhance bacterial translocation [34]. In all four groups of mice, none of the tissues showed changes in expression of the genes involved in the TLR pathway. In addition, our results from the histological, immunological and translocation analyses are consistent with the absence of inflammatory-response activation, or gut barrier dysfunction associated with the bacteroides intake.

Finally, the results of this study support our previous study indicating an anti-inflammatory effect of *B*. *uniformis* CECT 7771 *in vitro* in macrophages [3], as its administration to immunosuppressed mice also reduced the IFN-γ concentrations induced in the intestine. Administration of the bacteroide also reduced the *iNOS* expression, which was increased in the intestine and liver of immunosuppressed mice. The inducible isoform of nitric oxide synthases (iNOS) is one of three key enzymes generating nitric oxide (NO) from the amino acid L-arginine, which is involved in the immune response, acting as an immune defense mechanism and exerting cytotoxic effects at high concentrations. A variety of inflammatory stimuli (cytokines or bacterial pathogens) can activate iNOS expression, generating high concentrations of NO through the activation of inducible nuclear factors, including NFkB. For example inflammatory cytokine release (e.g. IFNγ and IL-1β) may activate NO production by altering the conformation of the *iNOS* promoter as in immunosuppressed mice where inflammatory cytokine levels are increased [35, 36]. In agreement with this mechanism, the administration of the bacteroide, exerting an anti-inflammatory effect, led to a reduction in both *iNOS* expression (gene and protein expression) and IFNγ production. Theoretically, LPS from Gram-negative bacteria, such as *Bacteroides* spp., could mediate activation of the TLR4 pathway, leading to MyD88-dependent activation of NF-κB and triggering a pro-inflammatory response as well as *iNOS* transcription to exclude pathogen invasion [35–38]. However, the capsular polysaccharide (PSA) of *Bacteroides fragilis* represents a reverse example, where the TLR detection of a microbial ligand via TLR2 promotes an anti-inflammatory response and immunologic tolerance of this bacterium by the host [39]. This could presumably be the case for *B*. *uniformis*, although the exact mechanism by which tolerance to this bacterium species is induced needs further investigations.

PPARγ gene and protein expression was also increased in the intestine of immunosuppressed mice. This was probably a protective response against the pro-inflammatory intestinal milieu caused by immunosuppression, which was also reduced by the bacteroides administration. In fact, PPARγ is recognized as a fundamental regulator of the immune response due to its ability to inhibit the expression of inflammatory cytokines and direct the differentiation of immune cells towards anti-inflammatory phenotypes. However, the administration of *B*. *uniformis* CECT 7771 reduced PPARγ overexpression in immunosuppressed mice, reaching similar levels to those found in controls. In this context, *Bacteroides thetaiotaomicron* has also been shown to antagonize transcription factor NF-kB and, thereby, exert an anti-inflammatory effect by enhancing the nuclear export of RelA, a subunit of the transcriptionally active NF-kB, facilitating its complex with PPARγ [40].

In conclusion, the results of this safety evaluation by acute oral administration of *B*. *uniformis* CECT 7771 to mice do not raise safety concerns. No adverse effects were observed regarding general health indicators, bacterial translocation, gut mucosal histology, organ function or immune markers. Moreover, *B*. *uniformis* CECT 7771 administration to immunosuppressed mice restored the levels of inflammatory cytokines and other immune regulatory genes and proteins. Further studies are required to confirm tolerability and safety in longer studies in rodents and, of course, in humans.
